# miRNA dysregulation in cancer: towards a mechanistic understanding

**DOI:** 10.3389/fgene.2014.00054

**Published:** 2014-03-18

**Authors:** Jayanth Kumar Palanichamy, Dinesh S. Rao

**Affiliations:** ^1^Department of Pathology and Laboratory Medicine, University of CaliforniaLos Angeles, CA, USA; ^2^Jonsson Comprehensive Cancer Center, University of CaliforniaLos Angeles, CA, USA; ^3^Broad Stem Cell Research Center, University of CaliforniaLos Angeles, CA, USA; ^4^Division of Biology, California Institute of TechnologyPasadena, CA, USA

**Keywords:** microRNAs, non-genetic heterogeneity, genetic redundancy, microRNA profiling, single-cell studies

## Abstract

It is now well known that gene expression is intricately regulated inside each cell especially in mammals. There are multiple layers of gene regulation active inside a cell at a given point of time. Gene expression is regulated post-transcriptionally by microRNAs and other factors. Mechanistically, microRNAs are known to bind to the 3’ UTR of mRNAs and cause repression of gene expression and the number of known microRNAs continues to increase every day. Dysregulated microRNA signatures in different types of cancer are being uncovered consistently implying their importance in cellular homeostasis. However when studied in isolation in mouse models, clear-cut cellular and molecular mechanisms have been described only for a select few microRNAs. What is the reason behind this discrepancy? Are microRNAs small players in gene regulation helping only to fine tune gene expression? Or are their roles tissue and cell type-specific with single-cell level effects on mRNA expression and microRNA threshold levels? Or does it all come down to the technical limitations of high-throughput techniques, resulting in false positive results? In this review, we will assess the challenges facing the field and potential avenues for resolving the cellular and molecular mechanisms of these small but important regulators of gene expression.

## BACKGROUND

microRNAs (miRNAs) are short single stranded RNAs that regulate mRNA expression at the post-transcriptional level. The very first miRNA:mRNA target pair was discovered in the labs of Victor Ambros and Gary Ruvkun (*lin-4* and *lin-14*, respectively) who were studying the regulation of developmental timing in *C. elegans *(Lee et al., 1993; Wightman et al., 1993). The mature miRNA is a single stranded ~22 nucleotide RNA that is sequentially processed from a primary transcript (pri-miRNA) and the resulting stem cell loop structure (pre-miRNA) by the proteins Drosha and Dicer respectively (Kim et al., 2009). The basic principle of miRNA mediated regulation- that miRNAs target mRNAs for repression by binding to the latter’s 3’UTR- was recognized in these early studies, but the pervasiveness of this mechanism in gene regulation was not recognized until much later. The number of miRNAs in humans and mice is constantly being revised, and it is likely that much of the genome is under the control of miRNA-mediated gene expression regulation (Kim et al., 2009).

Dysregulation of miRNAs has been observed in a number of diseases, especially hematopoietic malignancies like chronic lymphocytic leukemia (CLL) and epithelial malignancies such as lung cancers and breast carcinomas (Calin and Croce, 2006). However, the exact cause-effect mechanism has not been established for many miRNAs and diseases despite significant efforts into such research. Indeed, while numerous cancer-causing or promoting miRNAs have been proposed on the basis of their dysregulation in disease states, few have been established as truly oncogenic or tumor suppressive. Implicit in this observation is the question of whether we should be interpreting the roles of miRNAs in disease states in the same way we evaluate the role of proteins in oncogenesis, and what molecular and cell biological mechanisms distinguish miRNAs as novel factors in disease causation and progression. In this review, we will evaluate these questions critically, using hematologic cancers as an example. We speculate that the paucity of definitive experimental evidence is likely attributable to a combination of several factors- including, but not limited to, the inherent genetic redundancy of miRNAs, non-genetic heterogeneity of cells and our technical limitations in small RNA profiling. We begin with a brief primer on the complexity of miRNA based regulation and then examine research findings with the aim of understanding the limitations of current strategies to study miRNAs and exploring alternatives for the future.

## GENERATING COMPLEXITY: HOW THE BIOLOGY OF miRNAs IMPACTS INTERPRETATION OF PHENOTYPES

miRNA biogenesis and mechanism of action have been extensively studied and detailed mechanisms are reviewed elsewhere, but we review some of the mechanisms whereby complexity in gene regulation may be generated by miRNAs (Kim et al., 2009; Krol et al., 2010). The gene structure of miRNAs is the first source of complexity. Briefly, miRNAs can be encoded within an exon of a unique transcript, can occur as “polycistronic” miRNAs, or can be found within the introns of protein-coding or non-coding genes. The primary transcript undergoes endoribonucleolytic cleavage by Drosha/DGCR8 in the nucleus, and then in the cytoplasm. Intronic microRNAs (mirtrons) have also shown to be generated by a non-Drosha/DGCR8 mediated, Dicer only dependent mechanism (Castellano and Stebbing, 2013). Only one of the strands (the guide strand) thus generated is incorporated into the RNA induced silencing complex (RISC). Although the passenger strand is usually degraded, there are now reports emerging that show an important role for the passenger strand in gene regulation (Almeida et al., 2012; Epis et al., 2012; Uchino et al., 2013). The two strands of the microRNA are termed 5p and 3p depending on their orientation. Recent reports suggest that incorporation of either strand into the RISC complex is possible. Examples include miR-142 and miR-17 where incorporation of both the strands into the RISC complex occurs, leading to the presence of both 5p as well as 3p transcripts inside the cell (Kasashima et al., 2004; Yang et al., 2013). This introduces yet another layer of regulation since the 5p and 3p microRNAs would target different genes because of the difference in their seed sequence. This is the case with miR-28 where overexpression of miR-28-5p and miR-28-3p caused different effects in colorectal cancer cells (Almeida et al., 2012). Interestingly, miR-17-5p as well as miR-17-3p were shown to target the same gene TIMP3 and induced prostate cancer growth (Yang et al., 2013). All of these steps are subject to regulation and can therefore create intricate patterns of miRNA expression.

Once the miRNA is incorporated into RISC, it causes repression of target gene expression. miRNAs usually target the 3’UTR (untranslated region) of the mRNA. Perfect complementarity between the miRNA and its target leads to destruction of the RNA by the “slicer” activity in the Argonaute-2 protein which is part of the RISC (Meister, 2013). This type of repression is called RNAi and is very rare in mammals but more commonly seen in plants. More commonly, incomplete complementarity between the miRNA and its target is seen leading to translational repression. Proposed mechanisms include interference of the miRNA with the formation of a looped mRNA structure required for optimal translation, but further study is needed (Kim et al., 2009). Nonetheless, the net effect of miRNA expression is thought to be repression of the target gene. The repression is thought to be limited in extent (i.e., the gene is partially repressed) because miRNAs are induced and degraded over time.

Although binding to the 3’UTR and repression of the gene is considered to be the current canonical pathway for miRNA mediated gene regulation, non-canonical binding and targeting has also been described for microRNAs. Some microRNAs have been shown to target the promoters of genes and cause either gene activation (miR-373, miR-744, miR-1186) or gene repression (miR-320, miR-373). Gene activation was accompanied by the presence of active histone marks (H3K4 trimethylation) while gene repression was caused by a closed chromatin structure due to H3K9 dimethylation (Huang and Li, 2012). Recently, CLASH (cross-linking, ligation, and sequencing of hybrids) was used along with overexpression of an Ago1 fusion protein and high-throughput sequencing to map the miRNA-interactome. This revealed that even though the majority of the miRNA binding sites were in the 3’UTRs, microRNAs also bound to 5’UTRs, gene bodies, long non-coding RNAs (lncRNAs) as well as introns. There was also a class of microRNAs whose mRNA interactions did not involve the seed region (miR-92a), or so-called “seedless binding” (Helwak et al., 2013).

The temporal patterns of miRNA expression add significantly to the complexity of the problem. Some miRNAs are strongly induced by stimuli such as LPS (miR-155; O’Connell et al., 2009) whereas others are expressed at baseline levels and seem to maintain fairly constant levels during developmental processes (Pauli et al., 2011; Guan et al., 2013). In addition to their biogenesis, which can be regulated transcriptionally and post-transcriptionally at the level of RNA processing (van Kouwenhove et al., 2011), their degradation can be regulated both by proteins, as is the case with Xrn2 (Grosshans and Chatterjee, 2010), and by cell divisions, which dilute out the pool of miRNAs if their rate of production is less than the sum of dilution and degradation. As one considers disease-related changes in miRNA expression, understanding the temporal expression pattern, particularly whether phenotypes may be induced by various biological stimuli, impacts the interpretation of phenotypic changes. As an example, miR-155-deficient mice showed normal baseline levels of B-cells and T-cells, but these mice clearly had major defects in immune responses (Thai et al., 2007; O’Connell et al., 2010b; Huffaker et al., 2012). It is clear that miR-155 has major functions when induced, and that these functions have importance in the context of oncogenesis.

Adding to the complexity of miRNA mediated repression is computational and experimental evidence that a single miRNA can target hundreds or potentially thousands of mRNA molecules. The stoichiometry of such inhibition is incompletely understood, and it would seem that any two targets could be inhibited to different degrees by a given miRNA. Given computational algorithms that are imperfect and the lack of a methodology to predict the magnitude of repression, both the identity of and extent of effect on target gene expression have to be defined in an individual fashion by experimentation. Hence, it would be extremely difficult to predict the effects of miRNA expression on gene expression at the global level.

Post-transcriptional gene regulation by miRNAs is further modulated by RNA binding proteins (RBPs). Numerous RBPs have recently been characterized recently and many play a role in mRNA stability. A target mRNA can be competitively bound by a RBP and a miRNA, leading to a reduction in repression levels by the miRNA. RBP binding can also cause a switch in mRNA secondary structure, causing steric hindrance to miRNA binding. One example is the binding of IGF2BP1 (Insulin like growth factor 2 binding protein 1) to the *BTRC* and *MITF* mRNAs, preventing the binding of microRNAs 183 and 340 respectively. This leads to de-repression of these genes and subsequent downstream effects (Elcheva et al., 2009; Goswami et al., 2010; van Kouwenhove et al., 2011).

Lastly, there is a consideration about miRNA-based regulation that seems obvious but has important implications. miRNAs can only target transcripts that have been induced in a particular developmental or pathological context; they cannot, *a priori*, cause a gene to be expressed. Hence, the set of genes that a miRNA regulates in a given cell is highly dependent on the transcriptional program of that particular cell. Finding functional targets in one cell type does not necessarily implicate the same targets in a different cell type. Similarly, signaling pathways implicated in one cell type may not be operant in a different cell type. This has been highlighted by the germline knockout of miR-146a, which demonstrated a phenotype that was almost exclusively restricted to the hematopoietic system (Boldin et al., 2011; Zhao et al., 2011). This has important implications for how one predicts the effects of miRNA manipulation across different tissue types.. This also emphasizes the fact that microRNA targets will most probably be tissue type specific. So microRNA: mRNA interaction studies need to be performed specific to cell types and their results interpreted according to the tissue of origin.

## miRNAs AND CANCER: PROFILING STUDIES

After the recognition of miRNAs as being widespread in the genome of humans, there was a keen interest to identify dysregulated miRNA profiles in a number of diseases, especially cancer. Altered miRNA profiles were indeed found in many cancers (Calin and Croce, 2006). For example miR-21 was found to be up regulated in a number of malignancies like glioblastomas, breast, colon and pancreatic cancer and has been anointed as an oncogenic miRNA (Garzon et al., 2009). The miR 17–92 cluster was up regulated in lymphomas and in breast, lung, colon, stomach, and pancreatic cancers (Calin and Croce, 2009; Garzon et al., 2009).MiR-155 was found to be highly expressed in certain types of B-cell lymphomas, including CLL with an aggressive course and diffuse large B-cell lymphoma (Calin et al., 2004; Eis et al., 2005; Chen et al., 2008). Numerous other miRNAs have also been identified in patient-based samples as being dysregulated in disease states.

Similarly, some miRNAs are down regulated in tumors and are therefore classified as tumor suppressor miRNAs. The classic examples include miR-15a and miR-16-1 loss in CLL and multiple myeloma, let-7 miRNA loss in lung and breast cancers (Garzon et al., 2009). miR-34 is a p53 responsive miRNA family and its members, notably miR-34a, have been observed to be lost in pancreatic, colon, breast and liver cancers, and is a predictor of poor prognosis in CLL (Chim et al., 2010; Christoffersen et al., 2010; Corney et al., 2010; Hermeking, 2010; Choi et al., 2011; Fabbri et al., 2011). In myelodysplastic syndrome, miR-146a was downregulated, and at least part of the time, this occurs as a consequence of deletion of the long arm of chromosome 5 in the malignant hematopoietic progenitor cells (Starczynowski et al., 2010; Boldin et al., 2011; Zhao et al., 2013).

The last 10 years have seen a proliferation of miRNA profiling studies, and while many miRNAs are dysregulated at the level of their expression, it is unclear how miRNAs become downregulated in specific cancers. Until recently, the chromosomal deletion of miR-15a/16 (chromosome 12p) and miR-146a (chromosome 5q) comprised the only two recurrent genetic abnormalities that can be traced as a direct cause for miRNA downregulation in the hematopoietic system and as noted below, in cancer pathogenesis. Earlier last year, whole genome sequencing of *de novo* acute myeloid leukemia (AML) revealed that miR-142 undergoes point mutations, potentially resulting in loss of targeting by the 3p form (Cancer Genome Atlas Research Network, 2013). Further work has shown that miR-142 mutations in AML are localized to the seed region of miR-142-3p, leading to reduced expression of miR-142-5p. It is an interesting case where the stability of one form of the miRNA is dependent on the other form (Trissal et al., 2013). Other possible mechanisms include dysregulated miRNA expression resulting downstream of mutations in transcription factors. Since epigenetic studies of gene expression regulation have largely focused on protein-coding genes (where rules for histone modifications are better understood), annotations of miRNA promoters and enhancers have lagged behind those in the protein-coding genes. Therefore, further work in elucidating mechanisms of miRNA dysregulation and their integration into large scale genetic networks that are dysregulated in cancer is required.

## miRNAs: SMALL PLAYERS IN PATHOGENESIS?

As expected, altered miRNA profiles have been observed in numerous malignancies. However, there are only a few malignancies where a causal relationship has been directly established for the dysregulation of the miRNA and tumor development. A general role for the miRNA pathway in oncogenesis was established by the observation that mice heterozygous for Dicer developed an increased incidence of tumors (Kumar et al., 2009; Ravi et al., 2012). Hence, global abnormalities in miRNA expression are indeed causally linked to an increased incidence of tumors.

Among the well-known oncomiRs, overexpression of miR-21 in mice caused pre-B cell leukemia/lymphoma (Medina et al., 2010). In this study, miR-21 was expressed in a *Nestin-Cre*-inducible manner, surprisingly producing B-cell tumors. miR-155 overexpression led to a lymphoproliferative disorder in B-cells and eventually acute lymphoblastic lymphoma/leukemia (ALL), and myeloproliferative disease when overexpressed in myeloid cells (Costinean et al., 2006, 2009; O’Connell et al., 2011; Babar et al., 2012). Recently, overexpression of miR-22 in hematopoietic cells has been shown to lead to MDS (Song et al., 2013). Ectopic expression of miR-29a (Han et al., 2010) and enforced expression of miR-125b in hematopoietic stem cells (HSCs) led to a myeloproliferative disorder which progressed to AML (O’Connell et al., 2010a; Chaudhuri et al., 2012). Overexpression of the miR-17-19b cluster (five miRNAs) in the bone marrow of *Eμ-Myc *transgenic mice led to an acceleration of lymphoma development (He et al., 2005). Recently, B-cell specific overexpression of the miR 17–92 cluster led to lymphoma development, establishing its role as an independent oncogene (Jin et al., 2013). Other miRNAs which are highly expressed in tumors however, have failed to recapitulate the full tumor phenotype. For example, overexpression of the putative oncogenic miR-25-106b-93 cluster in mice showed no tumor development.

Deletions of tumor suppressor miRNA in mice have to date yielded phenotypes with a long latency. Deletion of the tumor suppressor miRNA cluster, encoding miR-15a and miR-16-1 led to a CLL like disease in mouse models, albeit at around 18 months of age and only in ~30% of the involved mice (Aqeilan et al., 2010; Klein et al., 2010). Deletion of the miRNA-146a led to a myeloproliferative phenotype and hematologic malignancies, again late in life (~18 months; Boldin et al., 2011; Zhao et al., 2011). Mice with targeted deletions of the following putative tumor suppressor miRNAs did not show development of an overt malignancy: miR-145, miR-223, miR-133, and miR-206 (Park et al., 2010). Although the overexpression of miR-29 had been shown to reduce liver cancer growth in mice (which is contrary to the role that it plays in the hematopoietic system), knockout of the miR-29 locus led to liver fibrosis and not any overt malignancy (Xiong et al., 2010; Roderburg et al., 2011). Perhaps the most surprising example of a knockout lacking a phenotype is the miR-34a knockout mouse, which did not develop any overt malignancies despite being downstream of arguably the most important tumor suppressor in the human cell, p53. Indeed, the triple knockout of all three members of miRNA family did not lead to an increase in cancer incidence (Concepcion et al., 2012). This is despite the fact that miR-34a targets numerous transcripts for proteins important in cell cycle progression, cell cycle progression, and oncogenesis (Siemens et al., 2013).

From a therapeutic standpoint, overexpression and *in vivo* delivery of miRNAs/ modified small nucleic acid molecules has met with some success. The overexpression of tumor suppressor miRNAs have been discovered to be successful in delaying tumor development or reducing the tumor burden in mouse models. Examples include miR-34a (lung adenocarcinoma), miR-145 and miR-33 (colon carcinoma) and miR-15 and 16-1 (colon carcinoma). Systemic delivery of the tumor suppressor let-7 increased the latency to lung cancer in mouse models (Croce, 2009; Nana-Sinkam and Croce, 2013).There are also some *in vivo* studies where knock down of oncomiRs has helped in attenuating tumorigenesis. In a mouse model of *Ras*-driven lung cancer, miR-21 inhibition slowed tumor progression (Hatley et al., 2010). Administration of antagomiRs in mice to block miR-10b and miR-223 resulted in significant antitumor activity against breast carcinoma and hepatocellular carcinoma respectively (Garzon et al., 2010).

In essence, not all dysregulated miRNAs discovered by expression profiling of tumors are pathogenetic and many do not have a clear mechanism of action in oncogenesis. In general, cancer phenotypes driven by overexpression seem to be more severe than those generated by loss-of-function, and the latter seem to be highly age dependent. These findings may be explained by the fact that miRNAs can only act on a “pre-existing” transcriptional program. Hence, miRNA gain of function in a cell type could lead to aberrant functions by targeting genes that are not normally repressed, while loss of function would only affect cells where the appropriate target genes and the miRNA of interest are concurrently expressed. This again brings forward the idea that miRNA gain and loss-of-function should be studied in the appropriate system and the appropriate biological context. One should also bear in mind the fact that microRNA overexpression or knockdown phenotypes have little utility in the absence of human disease specific dysregulation. Hence animal models and human studies should be used together to unravel the mechanism behind dysregulated miRNAs.

## miRNA PROFILING: USEFUL BUT NOT FOOLPROOF

Profiling of tumors provides patient-based data on miRNA expression patterns, which is undeniably important, but this presents its own set of problems. Centrally, it is very difficult to control for variability in collection of tumor cells between patients, as opposed to the ease of collecting high-quality controlled samples under experimental conditions. While it seems that miRNAs are better preserved between samples, as small RNAs demonstrate more stability than long RNAs, the techniques used to detect miRNAs also engender some variability. The common techniques for high-throughput profiling include microarrays, RNA-Seq and qPCR arrays. Cost and ease of data analysis remain the two main criteria which determine the type of platform utilized. The gold standard for individual miRNA detection remains the Northern blot and for samples with a low RNA yield (patient samples), reverse transcriptase (RT)-qPCR is the technique of choice for miRNA quantitation (Pritchard et al., 2012). However, interpretation of fold changes obtained from RT-qPCR experiments should always consider absolute levels of miRNA expression in control cells. For example, the biological impact of a 10-fold increase of a low expressed miRNA (1 molecule per cell increases to 10 molecules per cell) may be less significant than a 1.5-fold increase of a highly expressed miRNA (50,000 molecules per cell to 75,000 molecules per cell), depending on the relative expression levels of their mRNA targets. A last consideration in qPCR is the use of appropriate normalization techniques, investigators should consider the use of multiple reference genes or mean expression value of all expressed microRNAs in a sample as a normalization factor (Mestdagh et al., 2009).

Technically, both the RT step and the amount of RNA can be a source of significant bias during RNA profiling. A study comparing the efficiency of different types of RT enzymes showed around a 100-fold difference between enzymes for some genes (Stahlberg et al., 2004). This effect was particularly pronounced for mRNAs whose expression is low. Introduction of errors due to secondary structure, differences in priming efficiency and properties of the enzyme itself (like RNAse H activity) can all influence the product yield from the RT reaction. Also some miRNAs are GC rich and have a higher Tm which is a reason for reduced reverse priming efficiency and consequently smaller representation in the cDNA library. This effect will be much more pronounced when the quantity and quality of the RNA is low (for example, paraffin embedded samples). Similar to the RT reaction, each reaction in the sample preparation workflow may lead to variations including the adaptor ligation step using RNA ligase, linear amplification step as well as the fluorochrome labeling step for microarrays (Stahlberg et al., 2004; Baker, 2010).

With so many commercial options available for miRNA profiling, it is somewhat alarming that different groups have reported poor reproducibility across these different platforms. To avoid this problem, it has been suggested that once a certain profiling method has been standardized in the lab, maintaining the same technique is advisable to eliminate bias across methods (Git et al., 2010). Earlier quality control studies showed a big variation in the profiling of miRNAs by high-throughput sequencing especially when the quantity of transcripts was not abundant. However, encouragingly, recent reports on quality control of RNA-seq using newer kits suggests that current platforms are reproducible and comparable across each other (Adiconis et al., 2013).In addition to the range of platforms, data analysis can differ significantly between studies as well as labs, particularly in the stringency of false discovery rates and in the numerical level of difference between the samples. This too could contribute to the seemingly disparate results obtained by different groups.

Apart from these technical considerations, other factors may also lead to difficulty in fully understanding miRNA expression data. For example, gene duplications have led to miRNAs originating from different chromosomal loci, and each locus is subject to unique regulation. At present, we can only quantify the total amount of mature miRNA being produced because routine profiling studies do not distinguish which locus they originated from. Moreover, researchers have identified so-called IsomiRNAs, which are miRNAs that differ from their cognate miRNA by a few nucleotides either at the 3′ or even the 5′ end. The presence of IsomiRNAs could certainly cause a bias in mature miRNA quantitation by these high-throughput methods (Vickers et al., 2013). Hence, factors intrinsic to the roles of miRNAs in cells can cause variability in data generated by high-throughput studies.

Several proposals have been made to improve technical issues with miRNA based profiling techniques. Some laboratories have advocated the use of external reference samples for the exact quantitation of miRNAs (Git et al., 2010). But these reference samples do not mimic conditions inside the human body: most importantly, total RNA samples probably contain less than 0.01% miRNA (Git et al., 2010). There is ribosomal RNA, tRNA and mRNA that could compete for binding with preparative enzymes and thereby could potentially affect results. This is being negated to a certain extent now by removing the abundant ribosomal RNA before using the RNA for profiling (Git et al., 2010). It would be ideal if guidelines similar to the MIQE guidelines (minimum information for publication of quantitative real-time PCR experiments) were made available for miRNA profiling techniques (Bustin et al., 2013). This would ensure better experimental practice allowing more reliable comparative analysis of miRNA profiling data (Witwer, 2013). Hence, having a carefully curated database using uniform analytic techniques would go a long way toward removing obstacles that confound efforts to jump from profiling studies to functional analyses characterizing dysregulated miRNAs in cancer states.

Apart from high-throughput RNA sequencing, other novel techniques are also being employed to delineate targets of microRNAs. One of these is cross-linking immunoprecipitation-sequencing (CLIP-seq), where small RNAs and mRNAs bound to one of the proteins in the RISC, usually Ago-1, are cross-linked by ultraviolet radiation, pulled down using an anti-Ago1 antibody and then sequenced (Mittal and Zavolan, 2014). RISC-seq is a method similar to CLIP-seq using anti-Argonaute antibody pull-down followed by reverse transcription and sequencing. The difference here is the absence of any cross-linking (Matkovich et al., 2011). As mentioned previously, CLASH utilizes a similar cross-linking technique but ligates the miRNA and the mRNA using RNA ligase. While the specificity of CLASH is high, its sensitivity is reported to be very low (~2%; Helwak et al., 2013). The usage of these techniques in parallel with gene expression analyses in the setting of miRNA gain- or loss-of-function will likely increase the specificity of the observed microRNA targets.

## miRNAs AND REDUNDANCY

Here, we turn to some possible reasons as to why we see small phenotypes in miRNA deficient mice. The first of these relates to redundancy of both miRNAs and targeting of particular mRNAs by different miRNAs. In addition to there being multiple genomic loci producing the same miRNA, many miRNAs are part of miRNA “families,” which target the same seed sequence. Hence, there is a great deal of redundancy at the genomic level. Because the seed sequence is small, a single miRNA can target a large number of mRNAs (Thomson et al., 2011). The opposite is also true, that a single mRNA can be targeted by numerous miRNAs, including those from different families.

There are a number of miRNA target prediction tools available online including but not limited to TargetScan, PicTar, and Miranda (Ritchie et al., 2013). Confirmation of direct miRNA targeting of mRNA is usually done by a luciferase reporter-3′UTR of the mRNA construct and overexpressing the miRNA. However, not all of the predicted mRNA targets are actual targets of the miRNA. So what factors decide whether a miRNA will target a particular mRNA? There are many factors which likely play a role beyond seed matching and binding of complementary RNA strands. These may include structural accessibility of the 3′UTR of the mRNA to miRNAs, epigenetic modifications in targeted region preventing miRNA binding and the presence of miRNA sponges called competing endogenous RNAs (ceRNAs) which preferentially bind to miRNAs thereby de-repressing their mRNA targets (Karreth and Pandolfi, 2013). The prediction of miRNA-deficient phenotypes suffers from this lack of predictability of target repression.

Knocking out a single genetic locus encoding a miRNA may not be sufficient to fully understand the function of a given miRNA. Conceptually, this is similar to the situation of genetic redundancy seen with protein-coding genes, but it is different because miRNAs are most commonly found as families, and the majority of miRNAs share seed sequences with other microRNAs. Another important thing to note is the unperturbed levels of the miRNA in a particular tissue type. For example, miR-122 has been shown to account for nearly 50% of the total hepatocyte miRNA pool. In this situation, one could imagine that a knockout would have significant effects on gene expression (Szabo and Bala, 2013).

An ideal loss-of-function study will concurrently target all redundant miRNAs for deletion. This requires a large amount of resources to generate conventional knockout mice, which can be prohibitive to undertake such a project. However, with the advent of novel knockdown technologies like ZFNs (zinc finger nucleases), CRISPRs (clustered regularly interspaced short palindromic repeats) and TALENs (transcription activator-like effector nucleases), these complicated knockout animals may now be more feasible to create and study (Gaj et al., 2013). Alternative and novel approaches, such as designing ceRNAs that can target entire families of miRNAs, should also be considered and carefully examined. This approach could provide a viable method to knock down a large number of miRNAs at the same time. Lastly, the role of specific miRNA-target interactions should be carefully studied in carefully selected systems. In certain cases and in certain developmental/ pathologic contexts, a single miRNA-target pair may be of paramount importance (Xiao et al., 2007; O’Connell et al., 2009; Xiao and Rajewsky, 2009; Rao et al., 2010). Such interactions should be further studied using targeted mutations of the miRNA binding site in 3’UTRs, as described for miR-155 and AID (Borchert et al., 2011).

In most currently utilized techniques, the extent of overexpression is usually higher than physiologically attainable limits. Such overexpression may overwhelm the miRNA processing machinery and cause off-target effects (Singh et al., 2011; Karlsen and Brinchmann, 2013). Hence, some of the phenotypes that have been elaborated with overexpression may be more striking simply because of the extent of overexpression. Usage of endogenous promoters which are weaker than the conventional ones or using an inducible promoter which follows linear kinetics would be ideal, but this remains technically challenging. Yet, many of these experiments have revealed the central importance of miRNAs in driving oncogenesis.

## NON-GENETIC VARIATION AND THE THRESHOLD EFFECT

Recent studies have examined the role of variability of gene expression in the generation of different cell types during differentiation, and presumably similar mechanisms are operant in cancer causation and progression. Even when a tissue contains cells of the same type, the expression of a particular protein can show stochastic cell-to-cell as well as temporal fluctuations within the same cell. Sometimes these fluctuations were up to ~1000-fold between the low expressing and high expressing cells at one point of time (Chang et al., 2008). These differing gene expression levels might be purely stochastic or represent phenotypic variants within the same population (Mukherji et al., 2011). Recent reports suggest that such non-genetic heterogeneity might be generated by miRNA-mediated effects on gene expression, and that this may depend on the number of miRNA binding sites in the 3’UTR, the level of mRNA in the cell as well as the level of miRNA in the cell.

It has been demonstrated that at a constant level of miRNA, there is a particular threshold of mRNA below which the repression by the miRNA is dramatically increased (Mukherji et al., 2011). On the opposite end of the spectrum, past a certain concentration of mRNA, miRNA-mediated repression is virtually ineffective. Similarly when the number of binding sites in the 3’UTR for the miRNA was increased from 1 to 7 in the UTR, the repression increased by 40-fold (Chang et al., 2008). This threshold effect between the miRNA and the mRNA is reminiscent of biochemical precipitation reactions involving antigen- antibody interactions at the optimum concentration of both the antigen as well as the antibody. The precipitin line forms only at this zone of equivalence and neither in the region of antigen excess nor in antibody excess (Hornbeck, 2001). Hence, there are several relationships between the miRNA and mRNA that can profoundly change the distribution of gene expression within a population of cells.

**FIGURE 1:**
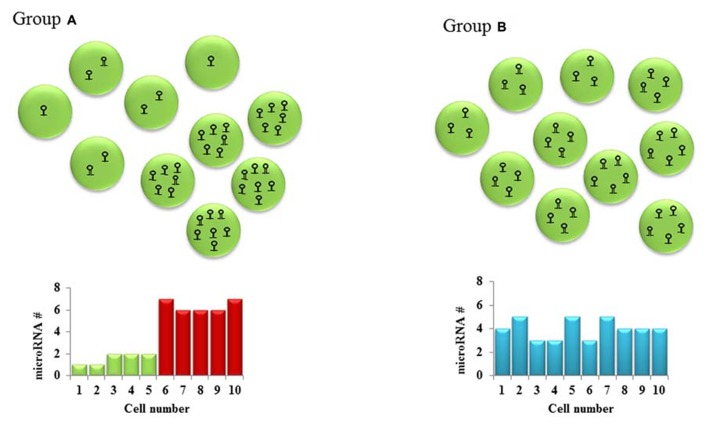
**Cellular heterogeneity in microRNA levels: Hypothetical cell groups (A,B) have the same average number of a particular microRNA per cell (average = 4)**. SNA extraction and qPCR of both these groups of cells would give the same microRNA expression in both groups. However, a closer look at the two groups shows a bimodal type of microRNA expression pattern in group **A** and a homogenous expression pattern in group **B**. This cell to cell difference in microRNA levels which might lead to different downstream gene expression is often overlooked in the absence of single-cell studies.

**FIGURE 2:**
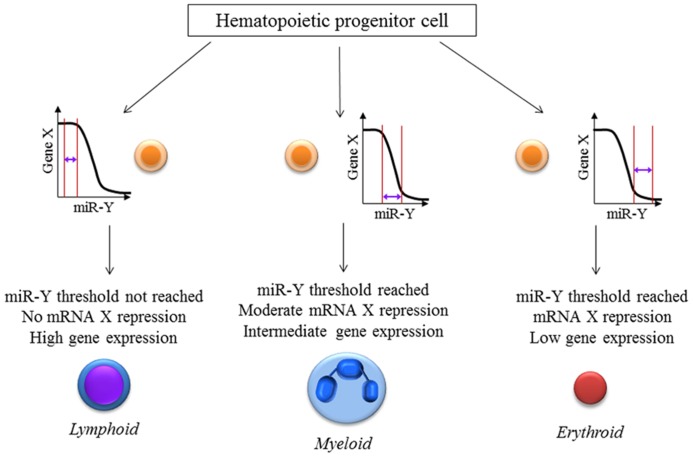
**miRNA:mRNA interaction-the threshold effect: The level of repression of gene expression is dependent on the levels of both the mRNA as well as the microRNA present inside a particular cell**. This hypothetical figure assumes that the expression of mRNA X is important for hematopoietic cell lineage determination with high, intermediate and low levels leading to lymphoid, myeloid and erythroid lineage differentiation respectively. Gene X is a known target of the microRNA miR-Y. These effects would be missed if single-cell measurements of X and miR-Y were not performed which reiterates the necessity for newer techniques and analyses especially at the single-cell level.

It is implied that a cell requires a particular level of mRNA and miRNA to coexist for repression to occur. The net effect of these threshold effects is that our conventional thinking about miRNA- that they partially repress gene expression- may only hold for intermediate concentrations of the miRNA and the target mRNA. The implications for experimental science are important: we have to be cognizant of the level of the target mRNA in the cells that are being targeted for perturbation of the miRNA. In experiments with miRNA overexpression or knock down, most groups usually collect the RNA from an entire population of cells, revealing a snapshot of the average gene expression in the cells. This certainly doesn’t tell us the entire story- different cells with differing unperturbed target mRNA levels would have different levels of change, depending on the mRNA threshold (**Figure 1**). Therefore, single-cell expression studies are necessary to study the cell to cell variation in response to miRNAs. Recent reports have shown that miRNA expression can be studied at the single-cell level using microfluidics (Wu et al., 2013). This, coupled with lineage tracing studies may help us understand how phenotypes develop in miRNA-deficient or overexpressing cells, both in development and in disease. The threshold effect is an important consideration in miRNA therapeutics- particularly against cancers. Overexpression of miRNAs might be highly efficient at repressing the expression of a critical target mRNA in cells that express the target at the optimum level (**Figure 2**). However cells with extremely high levels of the target mRNA would escape repression, if the above-proposed mechanisms are correct, and may even be selected for. In theory, this could lead to the elaboration of a more aggressive cancer following an initial remission. Hence, targeted therapy utilizing small RNA molecules requires careful consideration of ranges of target gene expression in the cancer cells.

## CONCLUSION

The plethora of profiling experiments has to date yielded important data about miRNA expression in primary human disease states, but we suggest that these constitute a starting point for disease-relevant studies. A major obstacle to understanding these miRNAs *in vivo* is the resource set required to individually target these miRNAs and miRNA-target pairs. However, it seems that such detailed studies will be required to understand the roles of these small RNAs in disease pathogenesis and for their utilization as therapeutic interventions.

We have tried to provide an overview of some of the factors that we believe to be obstacles in moving miRNA research from the bench to the bedside (or *vice-versa*). These include technical difficulties and variations in high-throughput profiling studies between labs and across platforms. These could be standardized in the future with rigorous quality control, better kits and standard guidelines for sample preparation and data analysis. The question of genetic redundancy in miRNAs calls for more complex mouse models which are also foreseeable in the future. Non-genetic heterogeneity presents a totally different question altogether. Sophisticated single-cell expression studies of mRNA, miRNA and proteins are required to pave the way to decipher the actual role of non-genetic variants present within what many researchers have previously presumed to be homogeneous populations. With the advent of more robust and reproducible profiling techniques, more sophisticated genetic manipulations in model systems, and single-cell analytical advances, we anticipate a clearer understanding of miRNA roles in biology and pathogenesis.

## Conflict of Interest Statement

The authors declare that the research was conducted in the absence of any commercial or financial relationships that could be construed as a potential conflict of interest.

## References

[B1] Adiconis X. Borges-Rivera D. Satija R. DeLuca D. S. Busby M. A. Berlin A. M. (2013). Comparative analysis of RNA sequencing methods for degraded or low-input samples. *Nat. Methods* 10 623–629 10.1038/nmeth.2483 23685885 PMC3821180

[B2] Almeida M. I. Nicoloso M. S. Zeng L. Ivan C. Spizzo R. Gafà R. (2012). Strand-specific miR-28-5p and miR-28-3p have distinct effects in colorectal cancer cells. *Gastroenterology* 142 886–896 10.1053/j.gastro.2011.12.047 22240480 PMC3321100

[B3] Aqeilan R. I. Calin G. A. Croce C. M. (2010). miR-15a and miR-16-1 in cancer: discovery, function and future perspectives. *Cell Death Differ.* 17 215–220 10.1038/cdd.2009.69 19498445

[B4] Babar I. A. Cheng C. J. Booth C. J. Liang X. Weidhaas J. B. Saltzman W. M. (2012). Nanoparticle-based therapy in an in vivo microRNA-155 (miR-155)-dependent mouse model of lymphoma. *Proc. Natl. Acad. Sci. U.S.A.* 109 E1695–E1704 10.1073/pnas.1201516109 22685206 PMC3387084

[B5] Baker M. (2010). microRNA profiling: separating signal from noise. *Nat. Methods* 7 687–692 10.1038/nmeth0910-687 20805796

[B6] Boldin M. P. Taganov K. D. Rao D. S. Yang L. Zhao J. L. Kalwani M. (2011). miR-146a is a significant brake on autoimmunity, myeloproliferation, and cancer in mice. *J. Exp. Med.* 208 1189–1201 10.1084/jem.20101823 21555486 PMC3173243

[B7] Borchert G. M. Holton N. W. Larson E. D. (2011). Repression of human activation induced cytidine deaminase by miR-93 and miR-155. *BMC Cancer* 11:347 10.1186/1471-2407-11-347 PMC3163633 21831295

[B8] Bustin S. A. Benes V. Garson J. Hellemans J. Huggett J. Kubista M. (2013). The need for transparency and good practices in the qPCR literature. *Nat. Methods* 10 1063–1067 10.1038/nmeth.2697 24173381

[B9] Calin G. A. Croce C. M. (2006). microRNA signatures in human cancers. *Nat. Rev. Cancer* 6 857–866 10.1038/nrc1997 17060945

[B10] Calin G. A. Croce C. M. (2009). Chronic lymphocytic leukemia: interplay between noncoding RNAs and protein-coding genes. *Blood* 114 4761–4770 10.1182/blood-2009-07-192740 19745066 PMC2786287

[B11] Calin G. A. Liu C. G. Sevignani C. Ferracin M. Felli N. Dumitru C. D. (2004). microRNA profiling reveals distinct signatures in B cell chronic lymphocytic leukemias. *Proc. Natl. Acad. Sci. U.S.A.* 101 11755–11760 10.1073/pnas.0404432101 15284443 PMC511048

[B12] Cancer Genome Atlas Research Network. (2013). Genomic and epigenomic landscapes of adult de novo acute myeloid leukemia. *N. Engl. J. Med.* 368 2059–2074 10.1056/NEJMoa1301689 23634996 PMC3767041

[B13] Castellano L. Stebbing J. (2013). Deep sequencing of small RNAs identifies canonical and non-canonical miRNA and endogenous siRNAs in mammalian somatic tissues. *Nucleic Acids Res*. 41 3339–3351 10.1093/nar/gks1474 23325850 PMC3597668

[B14] Chang H. H. Hemberg M. Barahona M. Ingber D. E. Huang S. (2008). Transcriptome-wide noise controls lineage choice in mammalian progenitor cells. *Nature* 453 544–547 10.1038/nature06965 18497826 PMC5546414

[B15] Chaudhuri A. A. So A. Y. Mehta A. Minisandram A. Sinha N. Jonsson V. D. (2012). Oncomir miR-125b regulates hematopoiesis by targeting the gene Lin28A. *Proc. Natl. Acad. Sci. U.S.A.* 109 4233–4238 10.1073/pnas.1200677109 22366319 PMC3306721

[B16] Chen L. Cui B. Zhang S. Chen G. Croce C. M. Kipps T. J. (2008). Association between the proficiency of B-cell receptor signaling and the relative expression levels of ZAP-70, SHIP-1, and Mir-155 in chronic lymphocytic leukemia. *Blood* 112 3155

[B17] Chim C. S. Wong K. Y. Qi Y. Loong F. Lam W. L. Wong L. G. (2010). Epigenetic inactivation of the miR-34a in hematological malignancies. *Carcinogenesis* 31 745–750 10.1093/carcin/bgq033 20118199

[B18] Choi Y. J. Lin C. P. Ho J. J. He X. Okada N. Bu P. (2011). miR-34 miRNAs provide a barrier for somatic cell reprogramming. *Nat. Cell Biol.* 13 1353–1360 10.1038/ncb2366 22020437 PMC3541684

[B19] Christoffersen N. R. Shalgi R. Frankel L. B. Leucci E. Lees M. Klausen M. (2010). p53-independent upregulation of miR-34a during oncogene-induced senescence represses MYC. *Cell Death Differ*. 17 236–245 10.1038/cdd.2009.109 19696787

[B20] Concepcion C. P. Han Y. C. Mu P. Bonetti C. Yao E. D’Andrea A. (2012). Intact p53-dependent responses in miR-34-deficient mice. *PLoS Genet*. 8:e1002797 10.1371/journal.pgen.1002797 PMC3406012 22844244

[B21] Corney D. C. Hwang C. I. Matoso A. Vogt M. Flesken-Nikitin A. Godwin A. K. (2010). Frequent downregulation of miR-34 family in human ovarian cancers. *Clin. Cancer Res.* 16 1119–1128 10.1158/1078-0432.CCR-09-2642 20145172 PMC2822884

[B22] Costinean S. Sandhu S. K. Pedersen I. M. Tili E. Trotta R. Perrotti D. (2009). Src homology 2 domain-containing inositol-5-phosphatase and CCAAT enhancer-binding protein beta are targeted by miR-155 in B cells of Emicro-MiR-155 transgenic mice. *Blood* 114 1374–1382 10.1182/blood-2009-05-220814 19520806 PMC2727407

[B23] Costinean S. Zanesi N. Pekarsky Y. Tili E. Volinia S. Heerema N. (2006). Pre-B cell proliferation and lymphoblastic leukemia/high-grade lymphoma in E(mu)-miR155 transgenic mice. *Proc. Natl. Acad. Sci. U.S.A.* 103 7024–7029 10.1073/pnas.0602266103 16641092 PMC1459012

[B24] Croce C. M. (2009). Causes and consequences of microRNA dysregulation in cancer. *Nat. Rev. Genet.* 10 704–714 10.1038/nrg2634 19763153 PMC3467096

[B25] Eis P. S. Tam W. Sun L. Chadburn A. Li Z. Gomez M. F. (2005). Accumulation of miR-155 and BIC RNA in human B cell lymphomas. *Proc. Natl. Acad. Sci. U.S.A.* 102 3627–3632 10.1073/pnas.0500613102 15738415 PMC552785

[B26] Elcheva I. Goswami S. Noubissi F. K. Spiegelman V. S. (2009). CRD-BP protects the coding region of betaTrCP1 mRNA from miR-183-mediated degradation. *Mol. Cell* 35 240–246 10.1016/j.molcel.2009.06.007 19647520 PMC2742352

[B27] Epis M. R. Giles K. M. Kalinowski F. C. Barker A. Cohen R. J. Leedman P. J. (2012). Regulation of expression of deoxyhypusine hydroxylase (DOHH), the enzyme that catalyzes the activation of eIF5A, by miR-331-3p and miR-642-5p in prostate cancer cells. *J. Biol. Chem.* 287 35251–35259 10.1074/jbc.M112.374686 22908221 PMC3471734

[B28] Fabbri M. Bottoni A. Shimizu M. Spizzo R. Nicoloso M. S. Rossi S. (2011). Association of a microRNA/TP53 feedback circuitry with pathogenesis and outcome of B-cell chronic lymphocytic leukemia. *JAMA* 305 59–67 10.1001/jama.2010.1919 21205967 PMC3690301

[B29] Gaj T. Gersbach C. A Barbas C. F. III. (2013). ZFN, TALEN, and CRISPR/Cas-based methods for genome engineering. *Trends Biotechnol*. 31 397–405 10.1016/j.tibtech.2013.04.004 23664777 PMC3694601

[B30] Garzon R. Calin G. A. Croce C. M. (2009). microRNAs in Cancer. *Annu. Rev. Med.* 60 167–179 10.1146/annurev.med.59.053006.104707 19630570

[B31] Garzon R. Marcucci G. Croce C. M. (2010). Targeting microRNAs in cancer: rationale, strategies and challenges. *Nat. Rev. Drug Discov.* 9 775–789 10.1038/nrd3179 20885409 PMC3904431

[B32] Git A. Dvinge H. Salmon-Divon M. Osborne M. Kutter C. Hadfield J. (2010). Systematic comparison of microarray profiling, real-time PCR, and next-generation sequencing technologies for measuring differential microRNA expression. *RNA* 16 991–1006 10.1261/rna.1947110 20360395 PMC2856892

[B33] Goswami S. Tarapore R. S. Teslaa J. J. Grinblat Y. Setaluri V. Spiegelman V. S. (2010). microRNA-340-mediated degradation of microphthalmia-associated transcription factor mRNA is inhibited by the coding region determinant-binding protein. *J. Biol. Chem.* 285 20532–20540 10.1074/jbc.M110.109298 20439467 PMC2898355

[B34] Grosshans H. Chatterjee S. (2010). microRNAses and the regulated degradation of mature animal miRNAs. *Adv. Exp. Med. Biol.* 700 140–155 10.1007/978-1-4419-7823-3_12 21627036

[B35] Guan D. Zhang W. Zhang W. Liu G. H. Belmonte J. C. (2013). Switching cell fate, ncRNAs coming to play. *Cell Death Dis.* 4:e464 10.1038/cddis.2012.196 PMC3563984 23328671

[B36] Han Y. C. Park C. Y. Bhagat G. Zhang J. Wang Y. Fan J. B. (2010). microRNA-29a induces aberrant self-renewal capacity in hematopoietic progenitors, biased myeloid development, and acute myeloid leukemia. *J. Exp. Med.* 207 475–489 10.1084/jem.20090831 20212066 PMC2839143

[B37] Hatley M. E. Patrick D. M. Garcia M. R. Richardson J. A. Bassel-Duby R. van Rooij E. (2010). Modulation of K-Ras-dependent lung tumorigenesis by microRNA-21. *Cancer Cell* 18 282–293 10.1016/j.ccr.2010.08.013 20832755 PMC2971666

[B38] He L. Thomson J. M. Hemann M. T. Hernando-Monge E. Mu D. Goodson S. (2005). A microRNA polycistron as a potential human oncogene. *Nature* 435 828–833 10.1038/nature03552 15944707 PMC4599349

[B39] Helwak A. Kudla G. Dudnakova T. Tollervey D. (2013). Mapping the human miRNA interactome by CLASH reveals frequent noncanonical binding. *Cell* 153 654–665 10.1016/j.cell.2013.03.043 23622248 PMC3650559

[B40] Hermeking H. (2010). The miR-34 family in cancer and apoptosis. *Cell Death Differ*. 17 193–199 10.1038/cdd.2009.56 19461653

[B41] Hornbeck P. (2001). Double-immunodiffusion assay for detecting specific antibodies. *Curr. Protoc. Immunol. Chap.* 2:Unit 2.3. 10.1002/0471142735.im0203s00 18432768

[B42] Huang V. Li L. C. (2012). miRNA goes nuclear. *RNA Biol*. 9 269–273 10.4161/rna.19354 22336708 PMC3384582

[B43] Huffaker T. B. Hu R. Runtsch M. C. Bake E. Chen X. Zhao J. (2012). Epistasis between microRNAs 155 and 146a during T cell-mediated antitumor immunity. *Cell Rep*. 2 1697–1709 10.1016/j.celrep.2012.10.025 23200854 PMC3628775

[B44] Jin H. Y. Oda H. Lai M. Skalsky R. L. Bethel K. Shepherd J. (2013). microRNA-17~92 plays a causative role in lymphomagenesis by coordinating multiple oncogenic pathways. *EMBO J*. 32 2377–2391 10.1038/emboj.2013.178 23921550 PMC3771343

[B45] Karlsen T. A. Brinchmann J. E. (2013). Liposome delivery of microRNA-145 to mesenchymal stem cells leads to immunological off-target effects mediated by RIG-I. *Mol. Ther.* 21 1169–1181 10.1038/mt.2013.55 23568258 PMC3677300

[B46] Karreth F. A. Pandolfi P. P. (2013). ceRNA cross-talk in cancer: when ce-bling rivalries go awry. *Cancer Discov*. 3 1113–1121 10.1158/2159-8290.CD-13-0202 24072616 PMC3801300

[B47] Kasashima K. Nakamura Y. Kozu T. (2004). Altered expression profiles of microRNAs during TPA-induced differentiation of HL-60 cells. *Biochem. Biophys. Res. Commun.* 322 403–410 10.1016/j.bbrc.2004.07.130 15325244

[B48] Kim V. N. Han J. Siomi M. C. (2009). Biogenesis of small RNAs in animals. *Nat. Rev. Mol. Cell Biol.* 10 126–139 10.1038/nrm2632 19165215

[B49] Klein U. Lia M. Crespo M. Siegel R. Shen Q. Mo T. (2010). The DLEU2/miR-15a/16-1 cluster controls B cell proliferation and its deletion leads to chronic lymphocytic leukemia. *Cancer Cell* 17 28–40 10.1016/j.ccr.2009.11.019 20060366

[B50] Krol J. Loedige I. Filipowicz W. (2010). The widespread regulation of microRNA biogenesis, function and decay. *Nat. Rev. Genet.* 11 597–610 10.1038/nrg2843 20661255

[B51] Kumar M. S. Pester R. E. Chen C. Y. Lane K. Chin C. Lu J. (2009). Dicer1 functions as a haploinsufficient tumor suppressor. *Genes Dev*. 23 2700–2704 10.1101/gad.1848209 19903759 PMC2788328

[B52] Lee R. C. Feinbaum R. L. Ambros V. (1993). The *C. elegans* heterochronic gene lin-4 encodes small RNAs with antisense complementarity to lin-14. *Cell* 75 843–854 10.1016/0092-8674(93)90529-Y 8252621

[B53] Matkovich S. J. Van Booven D. J. Eschenbacher W. H Dorn G. W. II. (2011). RISC RNA sequencing for context-specific identification of in vivo microRNA targets. *Circ. Res.* 108 18–26 10.1161/CIRCRESAHA.110.233528 21030712 PMC3017647

[B54] Medina P. P. Nolde M. Slack F. J. (2010). OncomiR addiction in an in vivo model of microRNA-21-induced pre-B-cell lymphoma. *Nature* 467 86–90 10.1038/nature09284 20693987

[B55] Meister G. (2013). Argonaute proteins: functional insights and emerging roles. *Nat. Rev. Genet.* 14 447–459 10.1038/nrg3462 23732335

[B56] Mestdagh P. Van Vlierberghe P. De Weer A. Muth D. Westermann F. Speleman F. (2009). A novel and universal method for microRNA RT-qPCR data normalization. *Genome Biol*. 10 R64 10.1186/gb-2009-10-6-r64 PMC2718498 19531210

[B57] Mittal N. Zavolan M. (2014). Seq and CLIP through the miRNA world. *Genome Biol*. 15 202 10.1186/gb4151 PMC4053862 24460822

[B58] Mukherji S. Ebert M. S. Zheng G. X. Tsang J. S. Sharp P. A van Oudenaarden A. (2011). microRNAs can generate thresholds in target gene expression. *Nat. Genet.* 43 854–859 10.1038/ng.905 21857679 PMC3163764

[B59] Nana-Sinkam S. P. Croce C. M. (2013). Clinical applications for microRNAs in cancer. *Clin. Pharmacol. Ther.* 93 98–104 10.1038/clpt.2012.192 23212103

[B60] O’Connell R. M. Chaudhuri A. A. Rao D. S. Baltimore D. (2009). Inositol phosphatase SHIP1 is a primary target of miR-155. *Proc. Natl. Acad. Sci. U.S.A.* 106 7113–7118 10.1073/pnas.0902636106 19359473 PMC2678424

[B61] O’Connell R. M. Chaudhuri A. A. Rao D. S. Gibson W. S. Balazs A. B. Baltimore D. (2010a). microRNAs enriched in hematopoietic stem cells differentially regulate long-term hematopoietic output. *Proc. Natl. Acad. Sci. U.S.A* 107 14235–14240 10.1073/pnas.1009798107 20660734 PMC2922591

[B62] O’Connell R. M. Kahn D. Gibson W. S. Round J. L. Scholz R. L. Chaudhuri A. A. (2010b). microRNA-155 promotes autoimmune inflammation by enhancing inflammatory T cell development. *Immunity* 33 607–619 10.1016/j.immuni.2010.09.009 20888269 PMC2966521

[B63] O’Connell R. M. Zhao J. L. Rao D. S. (2011). microRNA function in myeloid biology. *Blood* 118 2960–2969 10.1182/blood-2011-03-291971 21725054 PMC3175776

[B64] Park C. Y. Choi Y. S. McManus M. T. (2010). Analysis of microRNA knockouts in mice. *Hum. Mol. Genet.* 19 R169–R175 10.1093/hmg/ddq367 20805106 PMC2981466

[B65] Pauli A. Rinn J. L. Schier A. F. (2011). Non-coding RNAs as regulators of embryogenesis. *Nat. Rev. Genet.* 12 136–149 10.1038/nrg2904 21245830 PMC4081495

[B66] Pritchard C. C. Cheng H. H. Tewari M. (2012). microRNA profiling: approaches and considerations. *Nat. Rev. Genet.* 13 358–369 10.1038/nrg3198 22510765 PMC4517822

[B67] Rao D. S. O’Connell R. M. Chaudhuri A. A. Garcia-Flores Y. Geiger T. L. Baltimore D. (2010). microRNA-34a perturbs B lymphocyte development by repressing the forkhead box transcription factor Foxp1. *Immunity* 33 48–59 10.1016/j.immuni.2010.06.013 20598588 PMC2911227

[B68] Ravi A. Gurtan A. M. Kumar M. S. Bhutkar A. Chin C. Lu V. (2012). Proliferation and tumorigenesis of a murine sarcoma cell line in the absence of DICER1. *Cancer Cell* 21 848–855 10.1016/j.ccr.2012.04.037 22698408 PMC3385871

[B69] Ritchie W. Rasko J. E. Flamant S. (2013). microRNA target prediction and validation. *Adv. Exp. Med. Biol.* 774 39–53 10.1007/978-94-007-5590-1_3 23377967

[B70] Roderburg C. Urban G. W. Bettermann K. Vucur M. Zimmermann H. Schmidt S. (2011). micro-RNA profiling reveals a role for miR-29 in human and murine liver fibrosis. *Hepatology* 53 209–218 10.1002/hep.23922 20890893

[B71] Siemens H. Jackstadt R. Kaller M. Hermeking H. (2013). Repression of c-Kit by p53 is mediated by miR-34 and is associated with reduced chemoresistance, migration and stemness. *Oncotarget* 4 1399–1415 24009080 10.18632/oncotarget.1202 PMC3824539

[B72] Singh S. Narang A. S. Mahato R. I. (2011). Subcellular fate and off-target effects of siRNA, shRNA, and miRNA. *Pharm. Res.* 28 2996–3015 10.1007/s11095-011-0608-1 22033880

[B73] Song S. J. Ito K. Ala U. Kats L. Webster K. Sun S. M. (2013). The oncogenic microRNA miR-22 targets the TET2 tumor suppressor to promote hematopoietic stem cell self-renewal and transformation. *Cell Stem Cell* 13 87–101 10.1016/j.stem.2013.06.003 23827711 PMC3767186

[B74] Stahlberg A. Kubista M. Pfaffl M. (2004). Comparison of reverse transcriptases in gene expression analysis. *Clin. Chem.* 50 1678–1680 10.1373/clinchem.2004.035469 15331507

[B75] Starczynowski D. T. Kuchenbauer F. Argiropoulos B. Sung S. Morin R. Muranyi A. (2010). Identification of miR-145 and miR-146a as mediators of the 5q- syndrome phenotype. *Nat. Med.* 16 49–58 10.1038/nm.2054 19898489

[B76] Szabo G. Bala S. (2013). microRNAs in liver disease. *Nat. Rev. Gastroenterol. Hepatol.* 10 542–552 10.1038/nrgastro.2013.87 23689081 PMC4091636

[B77] Thai T. H. Calado D. P. Casola S. Ansel K. M. Xiao C. Xue Y. (2007). Regulation of the germinal center response by microRNA-155. *Science* 316 604–608 10.1126/science.1141229 17463289

[B78] Thomson D. W. Bracken C. P. Goodall G. J. (2011). Experimental strategies for microRNA target identification. *Nucleic Acids Res.* 39 6845–6853 10.1093/nar/gkr330 21652644 PMC3167600

[B79] Trissal M. Silva J. Wylie T. Hundal J. McGrath S. Magrini V. (2013). Dysregulation and recurrent mutation of miRNA-142 in *de novo* AML. *Blood* 122 472

[B80] Uchino K. Takeshita F. Takahashi R. U. Kosaka1 N. Fujiwara K. Naruoka H. (2013). Therapeutic effects of microRNA-582-5p and -3p on the inhibition of bladder cancer progression. *Mol. Ther.* 21 610–619 10.1038/mt.2012.269 23295946 PMC3589153

[B81] van Kouwenhove M. Kedde M. Agami R. (2011). microRNA regulation by RNA-binding proteins and its implications for cancer. *Nat. Rev. Cancer* 11 644–656 10.1038/nrc3107 21822212

[B82] Vickers K. C. Sethupathy P. Baran-Gale J. Remaley A. T. (2013). Complexity of microRNA function and the role of isomiRs in lipid homeostasis. *J. Lipid Res.* 54 1182–1191 10.1194/jlr.R034801 23505317 PMC3622316

[B83] Wightman B. Ha I. Ruvkun G. (1993). Posttranscriptional regulation of the heterochronic gene lin-14 by lin-4 mediates temporal pattern formation in *C. elegans. Cell* 75 855–862 10.1016/0092-8674(93)90530-4 8252622

[B84] Witwer K. W. (2013). Data submission and quality in microarray-based microRNA profiling. *Clin. Chem.* 59 392–400 10.1373/clinchem.2012.193813 23358751 PMC4037921

[B85] Wu M. Piccini M. Koh C. Y. Lam K. S. Singh A. K. (2013). Single cell microRNA analysis using microfluidic flow cytometry. *PLoS ONE* 8:e55044 10.1371/journal.pone.0055044 PMC3559333 23383050

[B86] Xiao C. Rajewsky K. (2009). microRNA control in the immune system: basic principles. *Cell* 136 26–36 10.1016/j.cell.2008.12.027 19135886

[B87] Xiao C. Calado D. P. Galler G. Thai T. H. Patterson H. C. Wang J. (2007). MiR-150 controls B cell differentiation by targeting the transcription factor c-Myb. *Cell* 131 146–159 10.1016/j.cell.2007.07.021 17923094

[B88] Xiong Y. Fang J. H. Yun J. P. Yang J. Zhang Y. Jia W. H. (2010). Effects of microRNA-29 on apoptosis, tumorigenicity, and prognosis of hepatocellular carcinoma. *Hepatology* 51 836–845 10.1002/hep.23380 20041405

[B89] Yang X. Du W. W. Li H. Liu F. Khorshidi A. Rutnam Z. J. (2013). Both mature miR-17-5p and passenger strand miR-17-3p target TIMP3 and induce prostate tumor growth and invasion. *Nucleic Acids Res*. 41 9688–9704 10.1093/nar/gkt680 23990326 PMC3834805

[B90] Zhao J. L. Rao D. S. Boldin M. P. Taganov K. D. O’Connell R. M. Baltimore D. (2011). NF-kappaB dysregulation in microRNA-146a-deficient mice drives the development of myeloid malignancies. *Proc. Natl. Acad. Sci. U.S.A.* 108 9184–9189 10.1073/pnas.1105398108 21576471 PMC3107319

[B91] Zhao J. L. Rao D. S. O’Connell R. M. Garcia-Flores Y. Baltimore D. (2013). microRNA-146a acts as a guardian of the quality and longevity of hematopoietic stem cells in mice. *Elife* 2:e00537 10.7554/eLife.00537 PMC3660742 23705069

